# #NoIDVape: A content analysis of illicit vape messaging in young people's information sources

**DOI:** 10.1111/add.70476

**Published:** 2026-05-31

**Authors:** Eleanor Bray, Sarah Gentry, Anna Varley, Caitlin Notley, Samuel Webb, Emma Ward

**Affiliations:** ^1^ Norwich Medical School University of East Anglia Norwich UK; ^2^ Independent public representative with lived experience in vaping UK

**Keywords:** digital health communication, educational resources, Illicit vaping, social media marketing, TikTok, underage access, youth vaping

## Abstract

**Background and aims:**

In recent years, the increased prevalence of youth vaping in the United Kingdom (UK) may have coincided with a proliferation in the use of ‘illicit’ (unregulated) vapes. Media or educational content about illicit vaping aimed at young people is scarce and poorly understood. This study aimed to systematically assess the content of both TikTok videos and educational resources relating to illicit vaping.

**Methods:**

A systematically conducted analysis of educational and social media resources. Manual searches were conducted in the UK to collect URLs and metadata for publicly available TikTok videos using eight illicit vaping‐related hashtags. Video content was coded across inductively derived thematic domains. Educational resources were collected via relevant search terms on Google; illicit vape content was appraised across five domains and the quality of each resource was assessed.

**Results:**

A total of 58 TikTok videos were categorised across nine thematic domains. The most prevalent themes were ‘Apathy Towards Law’ (57%), ‘Entertain/Humour’ (50%) and ‘Sub‐culture and Shared Experience’ (50%), generating a combined total of 21 million likes. Educational resources were rated as ‘good’ quality overall (56%) but underperformed in domains related to ‘Illicit Vapes Health Risk Depiction’ and ‘Relevance and Appeal to Youth’. In contrast to the ‘homemade’, ‘entertaining’ and sometimes ‘glamourising’ presentation of the TikTok videos, educational resources adopted a serious tone and were often disengaging.

**Conclusions:**

Illicit vaping content differs between TikTok videos and educational resources, exhibiting differences in sentiment, information and youth appeal. TikTok videos typically receive high engagement and frequently depict themes of apathy towards the law, entertainment and shared experience. Educational resources are generally of good quality but contain limited information on illicit vape health risks and lack youth relevance.

## INTRODUCTION

There has been an increase in the popularity of vaping among young people across industrialised countries, with usage rising considerably in recent years [1, 2, 3, 4]. According to the latest data from Action on Smoking and Health (ASH), 20% of young people 11 to 17 years old reported having tried vaping in Great Britain, with 7% identifying as current users [1]. This is a substantial increase compared to youth vaping rates in previous years, in which only 0.8% of young people were reported to be regular e‐cigarette users [2]. Such proliferation constitutes a potential growing public health concern and policymakers have warned that marketing vapes to ‘nicotine naive’ individuals could fuel addiction and function as a gateway to further nicotine use [5, 6, 7]. Studies typically define these individuals as having prior experience with nicotine or tobacco products or extremely limited exposure (typically a lifetime consumption of <20 tobacco cigarettes or e‐cigarettes).

Current legislation in the United Kingdom (UK) mandates that it is illegal to sell vaping products to individuals under the age of 18 [8]. Recent efforts to curb youth vaping include a ban on disposable vapes that came into force on the 1 June 2025. The recent Tobacco and Vapes Bill provides the UK government with powers to stop vapes from being strategically branded and advertised to appeal to children [9]. Although intending to address environmental impacts, the primary rationale underpinning these measures is to ‘tackle the rise in youth vaping and protect children's health’ [10] through reducing the availability and accessibility of disposable products.

Despite legislation prohibiting illicit activity, underage sales and purchases of vapes remain commonplace, such as older teenagers purchasing vapes online and on the high street for their younger peers [11]. Recent concerns have emerged around the wide availability of unregulated, ‘black market’ devices known as illicit vapes. In the UK context, illicit vapes refer to products that are non‐compliant with the Tobacco and Related Products Regulations (2016). Such products typically exceed permitted nicotine strengths (20 mg/ml maximum in the United Kingdom), have oversized tanks or lack appropriate health labelling or regulatory notification [12]. Counterfeit devices are often manufactured in unregulated environments and can be used as a tool to deliver illegal psychoactive substances such as Δ‐9‐tetrahydrocannabinol (THC) and Spice (a synthetic cannabinoid receptor agonist), posing additional harm to children and young people [13]. Since 2025, disposable vape sales have been prohibited in the United Kingdom and are now also considered illicit [14]. As regulations governing vaping products vary internationally, the term ‘illicit’ is used to broadly capture content promoting or depicting products that would be considered non‐compliant with UK regulatory standards.

The ubiquitous use of social media among young people has positioned video‐based platforms such as TikTok as a prominent medium for the dissemination and normalisation of vaping‐related content [15]. Vaping‐related content on TikTok garners substantial engagement and Sun *et al*. [3] found that 808 pro‐vaping videos collectively received over 1.5 billion views. Young people are routinely exposed to content that positively endorses vapes through themes of comedy, product promotion (i.e. novel colours and flavours) and vaping tricks, potentially fostering apathy toward nicotine addiction [3, 16, 17, 18]. Additionally, Jung *et al*. [19] analysed content under 50 vape‐related hashtags and found that vaping was depicted as a normalised aspect of daily life, with TikTok influencers subtly assimilating vaping into social practices and popular culture. Although important, these existing studies have focused on vaping more broadly, with content analyses rarely examining the portrayal of illicit products. The limited research examining illicit vaping content on TikTok is concerning, given evidence that creators frequently circumvent platform regulations by generating new hashtags to evade detection and promoting vaping products without age verification [20, 21].

In contrast, institutional educational resources typically frame vaping within a risk‐based narrative, often emphasising addiction and health concerns via prevention messaging [22]. There is a considerable dearth of research on the content of institutional resources containing illicit vape information [23], and these resources are rarely analysed alongside youth‐consumed social media content.

To our knowledge, no study to date has systematically compared how illicit vaping is framed in social media content versus institutional educational messaging. This comparison is important as young people's information exposure is increasingly influenced by algorithmically curated social media, rather than traditional educational materials. Research on young people's digital health information behaviours indicates that health promotion interventions and resources are still delivered via traditional websites, while young people rely on more novel video‐based platforms such as TikTok for information seeking [24, 25, 26]. Examining these divergent formats may illuminate a potential disconnect between the regulated, institutional messaging intended for young people and the content they are encountering in everyday digital spaces. This study aimed to systematically examine illicit vape content in TikToks and educational resources, highlighting communicative themes and considering the implications for youth‐facing health communication.

## METHODS

### Data collection

A content analysis was chosen as a relevant approach to explore both explicit information and any underlying narratives present in illicit vaping content. Manual searches were used as a method to simulate the typical user experience, as opposed to relying on algorithmically controlled datasets. Because of the diverse providers of educational resources (government agencies, non‐profit organisations, *etc*.), manual searches were deemed the most appropriate method for accessing widely available public resources that cannot be reliably captured through centralised databases and automated scraping.

#### Educational resources

**FIGURE 1:**
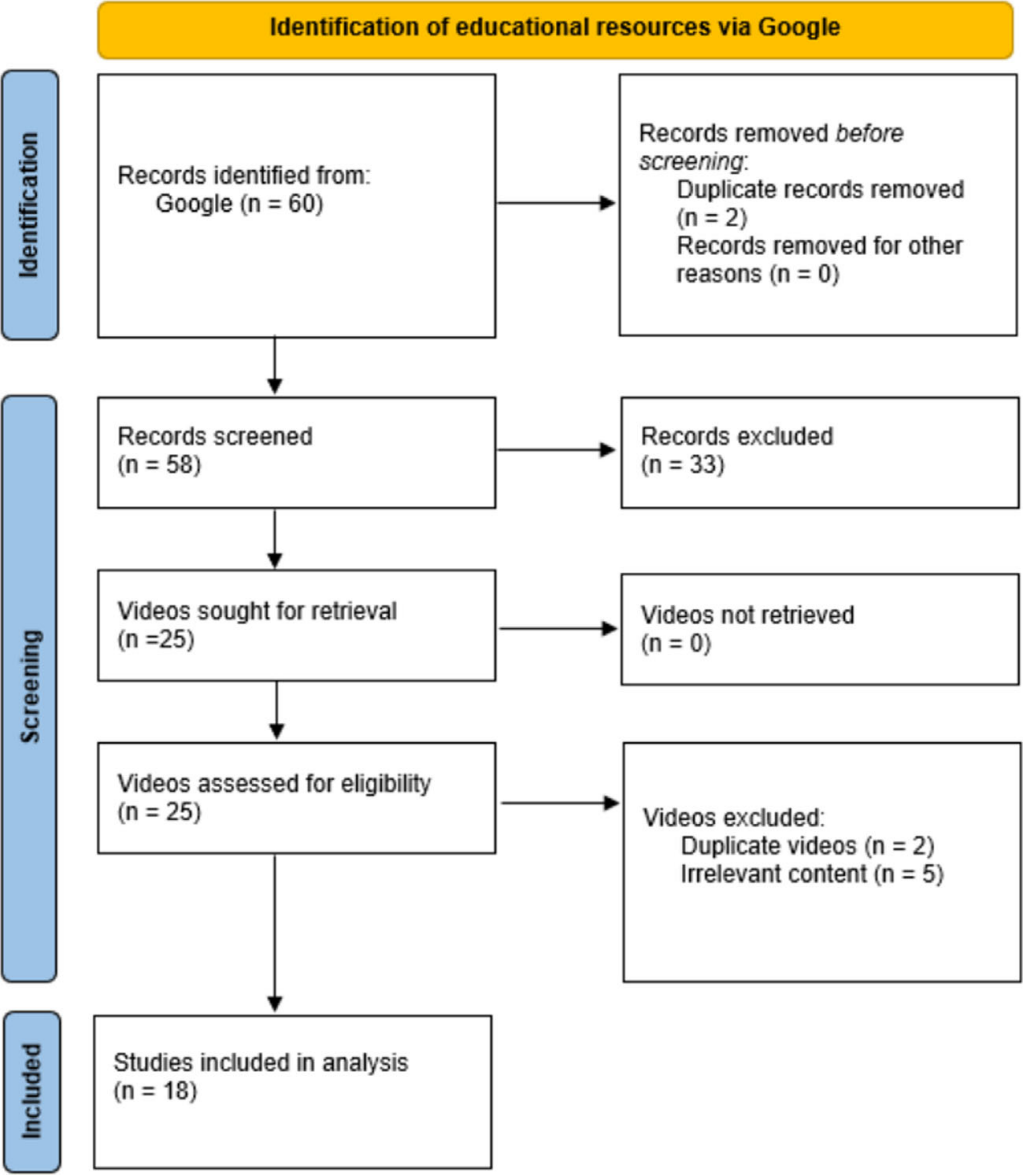
Preferred Reporting Items for Systematic reviews and Meta‐Analyses (PRISMA) flow style visual showing search methodology and resource selection.

Initial searches for educational resources were conducted in November 2024, using the Google search engine from a UK‐based IP address in incognito mode to minimise personalisation. The authors recognise the dynamic nature of Google search results and their implications for reproducibility and, therefore, provide a detailed documentation of the search strategy to enhance transparency. See Figure 1 for a Preferred Reporting Items for Systematic reviews and Meta‐Analyses (PRISMA) flow style visual detailing the screening process.

The search terms (‘educational resources’ and ‘illicit vapes’) were entered into the Google search engine, and after the removal of duplicates, the first six pages (58 Google search results) were manually screened for inclusion in the content analysis. Beyond the first six pages, websites were irrelevant and presented duplicates. Resources were included if they were publicly available, demonstrated reference to illicit vapes and were written in the English language. Those that did not meet the aforementioned criteria were excluded (i.e. resources behind a paywall). Following first level screening, two research team members (E.B., S.G.) used the exclusion and inclusion criteria to independently screen the resource titles and exclude any that were deemed irrelevant (*n* = 33), leaving 25 for full content review. Any discrepancies between screenings were resolved through dialogue and collaboration. During second level screening, the content of the resources was reviewed by the same two researchers and seven resources were excluded for not meeting the eligibility criteria. Of the total results screened, 18 educational resources met the criteria and were retained for analysis.

#### TikTok videos

A broad definition of illicit vaping was used in this study, including underage sales or purchase, underage use, vape concealment and non‐compliant products. As described in extant studies, a snowballing procedure produced 33 popular vaping‐related hashtags on TikTok in October 2024 [3, 27, 28]. This involved identifying relevant hashtags and observing further hashtags displayed within each of these to broaden the scope of search. The initial hashtags searched were generic and selected based on prior knowledge of TikTok content (i.e. ‘#vape’, ‘#vapelife’, ‘#juulgang’). Through further exploration, the hashtags were expanded by observing any that frequently appeared alongside or were auto‐suggested as ‘related’ to the original hashtags. Eight newly discovered hashtags were identified to ensure the searches returned the intended illicit vape‐related content: ‘#illegalvape’, ‘#discreetvapeshipping’, ‘#discreetpackaging’, ‘#noIDvape’, ‘#puffbundles’, ‘#lacedvape’, ‘#hiddennic’ and ‘#vapeshop.’

**FIGURE 2:**
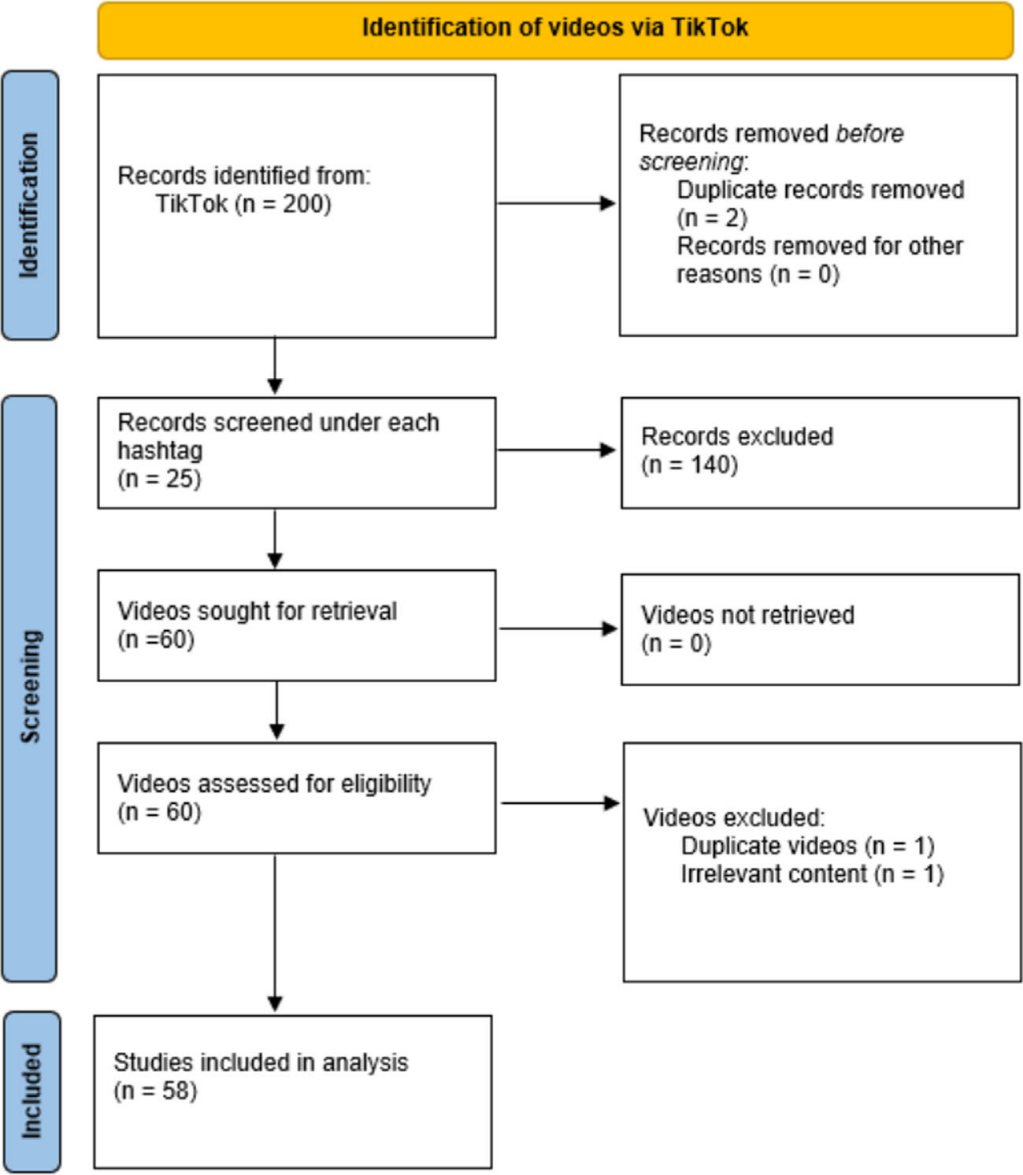
Preferred Reporting Items for Systematic reviews and Meta‐Analyses (PRISMA) flow style visual showing TikTok screening process.

Using a UK TikTok account created specifically for the study, a researcher (E.B.) downloaded 200 videos under the identified hashtags in November 2025. For each hashtag, the first 25 videos returned under TikTok's default ‘top’ ranking were screened for relevance. Videos were considered eligible if they were made in the English language and depicted or discussed illicit vape products according to the UK regulatory standards outlined previously. Videos made in non‐English languages in which dialogue was necessary for interpretation were excluded. A total of 60 videos were downloaded for closer review and after removal of duplicates and videos containing unrelated content, 58 videos remained in the final sample (see Figure 2). This search process was repeated on two different TikTok accounts. Screening continued until additional search results did not yield further videos meeting the eligibility criteria and results increasingly comprised unrelated content. Because of the researcher conducting the primary searches being female, there is a potential that TikTok's algorithm may tailor search results based on perceived gender‐related behaviours. Therefore, to mitigate any potential gender biases, we recruited S.W. as a male young person with lived experience of vaping as a Patient and Participant Involvement and Engagement (PPIE) representative to repeat the aforementioned search process on a separate UK account.

### Data analysis

National Health Services (NHS) Ethical approval was not required because of human subjects not being directly recruited for involvement in this project. All secondary source data is publicly available online and on TikTok. Following British Psychological Society guidelines for internet‐mediated research, informed consent was not considered necessary because of the public nature of the data [29]. To protect the anonymity of the content creators, any identifiable information was redacted and facial features were blurred.

#### Codebook development for educational resources

We used the data extraction form developed by Chen *et al*. [30] for an analysis of online electronic cigarette information, and the Quality Evaluation Scoring Tool (QUEST) [31] as a basis for the development of our data extraction and educational resource quality appraisal form. As this prior tool was not specifically designed to evaluate illicit vaping information, domains were refined through iterative review of the included resources. Through a combination of consulting the literature and repeated review of the content, particular categories were adjusted to capture content related to illicit vape health risks, regulatory issues and relevance to young audiences. In cases where overlapping categories were identified, domains were merged or refined following discussion among the research team. The framework was considered finalised once consensus was reached and domains adequately reflected the resource content.

Our data extraction form included: educational resource characteristics (including source, country of origin, resource title and target audience), main messages regarding illicit vapes and website credibility. Website credibility was assessed using five equally weighted criteria: authorship, quality of evidence, health risk depiction of illicit vapes, social responsibility and relevance and appeal to youth. Each resource was given a score of between 1 and 5 with higher scores indicating better quality. A mean score was calculated and resources were rated as ‘excellent’, ‘good’, ‘satisfactory’, ‘poor’ and ‘very poor.’ One author (E.B.) extracted information from included websites, and a second author (S.G.) checked the data extraction. Any differences were resolved through discussion.

S.W. helped code the ‘relevance/appeal to youth’ category, to validate coding decisions and reduce researcher bias in interpreting what is most engaging and relatable to younger audiences. Each individual educational resource was shown to S.W., providing an opportunity to verbally discuss their interpretation and decide on a rating with E.B.

#### TikTok videos

Mirroring approaches undertaken in previous TikTok analyses, A.V. and E.W. independently inductively coded a subsample (*n* = 43) of videos thematically (in Microsoft Excel) and met to compare their coding and resolve any inconsistencies [32, 33]. This inductive analysis informed the inclusion and labelling of the thematic domains and subdomains used for the codebook. The resulting codebook sought to classify the videos across nine thematic domains including: (1) content type; (2) depiction type; (3) concealment and accessibility; (4) marketing and sales; (5) social and cultural perceptions; (6) health and environment; (7) law and legislation; (8) alarm and advocacy; and (9) illicit and counterfeit products. The codebook was tested on a subsample of videos by S.G. and A.V. and coded data was populated on an Excel spreadsheet. One additional thematic subdomain was added after this process (‘content of vapes’).

Two independent coders (A.V. and S.G.) applied the coding framework to all videos within the dataset. All videos were binary coded; if a theme was detected in a video (i.e. promotional content relating to marketing and sales of illicit vapes), it was coded as ‘1’, if not present, it was coded as ‘0’. The domains were not mutually exclusive and although they are presented as separate entities, videos could be classified into multiple domains. A video was classified as depicting illicit vape content if it met one of the following criteria: mentioning of or visually promoting a non‐compliant device; endorsing underage sales or purchasing; promoting strategies to circumvent legal restrictions; or providing tips for concealing devices. In cases where videos presented ambiguous content, coders discussed the material and reached a consensus classification. Intercoder reliability was assessed using Cohen's κ on a random subset of 20% of videos, indicating substantial agreement across themes (κ = 0.78).

Several videos had been removed or deleted by the time the coding process commenced. This was likely because of the presence of illicit content prohibited by TikTok's policies. In these cases, videos were coded based on detailed descriptions and summaries (written by E.B. on extraction). To ensure consistency, coding discrepancies were resolved through dialogue and a further member of the research team (E.B.) inspected codes and made an executive decision for any conflicts.

The analytical write‐up alongside a sub‐sample of representative TikToks was shared with S.W. to conduct a PPIE ‘sense check’ of the findings. This enabled the young person to assess whether results aligned with youth experiences, while identifying any misinterpretations and providing feedback to improve the relevance and impact of findings [34]. This study was not pre‐registered, therefore, the analyses should be considered exploratory.

## RESULTS

### Educational resources

A total of 18 educational resources were identified and their main characteristics and their overall quality are presented narratively and in Table 1.

**TABLE 1:** Main characteristics and quality scores of educational resources.

Name of resource	Country	Resource provider	Type of resource	Intended audience	Authorship	Quality of evidence/accuracy of information	Illicit health risks depiction	Social responsibility	Relevance/appeal to youth	Overall quality rating
Vaping: The Facts (Smokefree Sheffield)	UK	Government/public health organisation	Campaign	CYP	5	4	2	5	4	Good
‘Vape’ (FRANK)	UK	Government/public health organisation	Digital information guide	CYP	5	4	3	5	5	Good
‘Vaping’ (Oak National Academy)	UK	Non‐profit health/education organisation	Educational presentation	Schools	4	3	2	2	2	Poor
‘Catch Your Breath’—The Smoking and Vaping Programme for Schools (Healthy Schools Cambridgeshire and Peterborough)	UK	Local council support service	Digital learning programme	Schools	4	5	3	5	4	Good
‘The facts of Vaping’ (Vaping Facts)	NZ	Government/public health organisation	Website	CYP	5	5	4	5	4	Good
Jordan North: How Safe is Vaping for my Health? (BBC News)	UK	News/broadcast provider	News article	General public	3	4	3	4	2	Satisfactory
Addressing Common Myths About Vaping (ASH)	UK	Government/public health organisation	Public health brief	General public	5	5	3	4	2	Satisfactory
Vaping Myths and the Facts (NHS, Better Health)	UK	Government/public health organisation	Digital information guide	General public	5	4	2	4	4	Good
Protect Your Breath (Te Whatu Ora, NZ)	NZ	Government/public health organisation	Campaign	CYP	4	5	3	5	5	Good
E‐Cigarettes: Facts, Stats and Regulations (Truth Initiative)	US	Non‐profit health/education organisation	Digital information guide	General public	5	4	2	5	4	Good
Youth Vaping Prevention (Truth Initiative)	US	Non‐profit health/education organisation	Digital learning programme	CYP	5	5	2	5	5	Good
Vaping: A Guide for Young People and Parents (Catch 22)	UK	Non‐profit health/education organisation	Digital information guide	CYP; parents	3	3	3	3	3	Satisfactory
Teaching About Vaping (Votes for Schools)	UK	Non‐profit health/education organisation	Digital learning programme	Schools	4	5	3	2	3	Satisfactory
Vaping Education Campaign (Trading Standards Service)	UK	Non‐departmental public body	Digital information guide	General public	5	4	3	4	2	Satisfactory
Youth Vaping (Safer Schools)	UK	Non‐profit health/education organisation	Digital information guide	Schools	4	4	4	5	3	Good
E‐Cigarette and Vaping Resources (Youth Now)	UK	Non‐profit health/education organisation	Digital learning programme	Schools	5	3	3	5	4	Satisfactory
Young People and Vaping (Gloucestershire Healthy Living and Learning)	UK	Local council support service	Digital information guide	General public	5	3	3	5	2	Satisfactory
What are Illegal Vapes (No Ifs. No Butts.)	UK/Wales	Non‐profit health/education organisation	Digital information guide	General public	4	4	4	4	4	Good
Key										
Excellent										
Good										
Satisfactory										
Poor										
Very poor										

Abbreviations: ASH, Action on Smoking and Health; BBC, British Broadcasting Corporation; CYP, children and young people; NHS, National Health Services; NZ, New Zealand; UK, United Kingdom.

#### Main characteristics of resources

The majority of the online resources were produced in the United Kingdom (72%) and were provided by non‐profit health or education organisations (44%). Digital information guides and webpages were the most common form of educational resources among those returned (50%), followed by information contained within campaigns (17%). The overall quality assessment rated zero as ‘excellent’, 56% of the educational resources as ‘good’, 39% as satisfactory, 6% as ‘poor’ and zero as ‘very poor’.

With regards to content coverage (Table 2), a high proportion of resources provided an overview of vapes/vaping (89%), however, those resources including a detailed discussion of illicit vaping was much less (63%). The most prevalent concepts mentioned in relation to illegal vapes were dangerous/hazardous chemicals, ease of access to illegal products, underage sales and legislation relating to the sales of illegal vapes. However, this information was often brief and presented as a piece of ‘add on’ information. There was limited information on how to detect an illegal vape, with only one resource ‘What are Illegal Vapes (No Ifs, No Butts)’ explicitly including a ‘How to Spot it?’ section. This included information on how to assess the legitimacy of a vape such as non‐compliant packaging and excessive puff counts. Vaping regulation was a particularly common content area (95%), with many providing information on current laws and age restrictions as well as vapes being presented as a smoking cessation method (84%).

**TABLE 2:** Content coverage of educational resources.

Content coverage	Educational resources (*n* = 18), *n* (%)
Overview of vapes/vaping	17 (89)
Health impact of vapes	18 (100)
Environmental impact of vapes	4 (21)
Use of vapes for smoking cessation	16 (84)
Regulation on vapes	18 (95)
Illicit vape coverage	12 (63)

*Note*: ‘Illicit vape coverage’ refers to the comprehensive coverage of illicit vape information.

#### Quality assessment of educational resources

A high performing category was ‘social responsibility’ in which 53% of resources were rated excellent (Table 3). As such, most resources promoted responsible behaviour and public health awareness through providing links to further resources or support services (i.e. ‘This is Quitting’ text message service). Furthermore, 42% of resources were rated both ‘good’ and ‘excellent’ for ‘authorship’ with many coming from reputable and established sources such as ASH. The majority of resources rated ‘excellent’ (33%) and ‘good’ (42%) in terms of the ‘quality of evidence’ presented. However, 22% were deemed only ‘satisfactory’ with content often including inaccurate information, sensationalist health claims (i.e. ‘vaping can result in heart attack or death’) and insufficient citations.

**TABLE 3:** Quality criteria and scores for each category.

Quality criteria	Educational resources (*n* = 18), *n* (%)
Authorship	
Excellent	8 (42)
Good	8 (42)
Satisfactory	2 (11)
Poor	0 (0)
Very poor	0 (0)
Quality of evidence/accuracy of information	
Excellent	6 (33)
Good	8 (42)
Satisfactory	4 (22)
Poor	0 (0)
Very poor	0 (0)
Illicit vapes health risks depiction	
Excellent	0 (0)
Good	3 (17)
Satisfactory	10 (56)
Poor	4 (22)
Very poor	0 (0)
Social responsibility	
Excellent	10 (53)
Good	5 (26)
Satisfactory	1 (6)
Poor	2 (11)
Very poor	0 (0)
Relevance/appeal to youth	
Excellent	2 (11)
Good	7 (29)
Satisfactory	3 (17)
Poor	5 (28)
Very poor	0 (0)

Quality ratings for the ‘illicit health risk depictions’ category were heterogeneous with zero resources scoring ‘excellent’, 17% scoring ‘good’, 56% scoring ‘satisfactory’ and 22% scoring ‘poor’. Higher‐rated resources provided a sufficient overview of the dangers associated with illicit vapes (i.e. the presence of potentially hazardous constituents, the carcinogenic properties of prohibited chemicals and their implications for broader disease risk). In addition, these resources drew a clear distinction between the health risks of regulated devices compared to unregulated devices. As such, they outlined the addictive nature of nicotine contained within regulated devices, but made clear their critical function in vaping cessation for adults and their comparatively lower health harms relative to smoking.

Resources scoring higher in this category highlighted that the harmful substances found in illicit products such as lead, nickel and chromium can have serious health impacts and implications in vaping‐related disorders. A substantial proportion (56%) of resources rating ‘satisfactory’ briefly mentioned the critical issues surrounding illicit vapes such as prohibited chemicals, underage sales and the legality surrounding e‐liquid nicotine concentration. However, these resources lacked any detailed discussion on the health impacts of using illegal devices and claims were not sufficiently cited. Direct references to any health information surrounding illicit vapes and their potentially dangerous constituents were absent in 22% of resources. Here, the resources primarily discussed the legality of vapes and their role in smoking cessation, but presented no illicit vaping‐related health risks.

Quality scores for the ‘relevance and appeal to youth category’ showed high dispersion with 11% rating ‘excellent’, 29% rating ‘good’, 17% rating ‘satisfactory’ and 28% rating ‘poor’ (as indicated by S.W., the PPIE representative). Higher rated resources within this category presented information clearly and concisely, using colour, animations and interactive features such as collapsible tabs and hyperlinks. The use of questions to guide the reader (i.e. ‘Did you know?’) was noted as a particularly engaging element. These resources provided pragmatic information, avoided jargon and used youth dialect such as ‘red flags’ to facilitate engagement. Those rated poorly were characterised by an overload of information often through dense ‘walls of text’ and had limited visual engagement. Some tended to be more legislation‐oriented, relying on oversimplified directives such as ‘If you are a child, do not vape’, which were deemed irrelevant and ineffective as a form of health communication.

### TikTok videos

After removing duplicates and videos that did not meet the inclusion criteria (*n* = 38), a final sample of 58 videos was yielded. The frequency of videos occurring under each domain and their collective number of likes are presented in Table 4 below. The like count was recorded as of the 20 February 2025, meaning each like count value is likely to have changed since the original date.

**TABLE 4:** Summary of key results and associated definitions used for coding depiction type and domains.

	Definition	Number of videos (*n* = 58, *n* = %)	Collective number of ‘likes’	Median ‘likes’ per video
Content type				
Inform/educate	Videos containing information about the risks, legality/regulation or health effects of illicit vapes (including ingredients)	17 (29)	1 742 552	4763
Promote/market	Videos advertising or promoting illegal vapes, often with links or calls to action for purchase	20 (34)	1 359 891	251
Entertain/humour	Videos promoting humorous, satirical or otherwise entertaining content related to illicit vapes	29 (50)	8 849 847	251
Testimonial/review	Videos including personal stories or reviews about using illegal vapes	9 (15)	71 969	99 000
Depiction type				
Positive	Promoting/encouraging illicit vape use	27 (47)	4 938 628	251
Negative	Discouraging/deterring illicit vape use including exposing sellers or warning viewers about illegal vape dangers	13 (22)	2 247 561	1027
Neutral	Ambiguous/no clear stance on illicit vape use	18 (31)	4 409 196	45 300
Concealment and accessibility				
Vape concealment and evasion strategies	Content concealing vaping from authority figures (e.g. parents or teachers) with tips or humour	23 (40)	1 712 831	1027
Discreet packaging	Content depicting packaging of vape products designed to avoid detection (i.e. everyday object disguises, or misleading labels to obscure the product's nature)	12 (21)	349 698	155
Underage sales/purchasing	Content depicting, referencing or normalizing the sale or purchase of vape products by individuals below the legal age	28 (48)	6 188 814	45 300
Ease of access	Content highlighting the simplicity of obtaining vape products, including minimal age verification, widespread availability or convenient purchasing options online or in‐store	28 (48)	3 508 830	4315
Marketing and sales				
Illicit online sales	Content depicting the unauthorized or illegal sale of vape products online, often involving unregulated platforms, private transactions or strategies to evade legal restrictions	22 (38)	80 906	251
Romanticisation of vapes	Content portraying vaping in an idealized or glamorous way, emphasizing its appeal as trendy, sophisticated or socially desirable while downplaying associated risks	21 (36)	746 305	53 200
Appealing marketing	Content showcasing vape products in a way designed to attract users, often through vibrant visuals, enticing flavours, influencer endorsements or themes that resonate with younger audiences	14 (24)	953 510	8360.5
Social and cultural perceptions				
Apathy towards law	Videos reflecting indifference or disregard for vaping regulations or laws, often trivializing enforcement or promoting behaviours that openly defy legal restrictions	33 (57)	6 476 097	38 600
Social responsibility and moral discourse around vaping	Content calling for accountability from individuals, businesses or society in regulating vaping behaviour; framing vaping as a moral issue or public concern; could also include videos mocking or rebutting social responsibility and moral discourse	18 (31)	2 605 284	18 454
Normalisation of vaping	Content that portrays vaping as a common, acceptable or unremarkable behaviour, reducing its perceived risks and making it seem like a socially acceptable or routine activity; could also include videos commenting on the negative impact of normalisation	23 (40)	5 062 347	45 300
Satisfaction from rule breaking	Content that portrays rule‐breaking (e.g. underage vaping) as satisfying or thrilling	21 (36)	24 517 530	68 100
Subculture and shared experience	Content that portrays vaping as part of a distinct social group or subculture, emphasizing a sense of belonging, shared identity or common experience	29 (50)	6 107 176	16 300
Health and environment				
Vaping Dependence (i.e. nicotine and addiction)	Content that discusses or depicts the physical or psychological reliance on vaping, often focusing on nicotine addiction, withdrawal symptoms, and the challenges of quitting or managing dependence	6 (10)	649.698	3166
Vaping for mental health	Content that portrays vaping as a coping mechanism for managing stress, anxiety, depression or other mental health challenges, often highlighting the use of vaping to relieve emotional or psychological distress	2 (3)	634 708	317 354
Health concerns of illicit vapes	Content that highlights the potential health risks associated with using unregulated or counterfeit vape products, including harmful ingredients, lack of quality control, and the unknown long‐term effects on physical health	11 (19)	1 391 298	2017
Vaping cessation	Content that focuses on quitting or reducing vaping, including strategies, personal stories or resources aimed at helping individuals overcome nicotine dependence and stop using vape products	3 (5)	639 023	4315
Environmental effects of illicit vapes	Content that discusses the ecological impact of unregulated or counterfeit vape products, including e‐waste, battery disposal issues, plastic pollution and the lack of proper recycling options	1 (2)	195	195
Law and legislation				
Vape legislation	Content that discusses laws, regulations or policies related to the sale, use and marketing of vape products, including age restrictions, product standards and government efforts to regulate the vaping industry	18 (31)	2 696 290	8407
Age restrictions	Content that discusses the legal age limits for purchasing and using vape products, including enforcement challenges, compliance issues or the impact of these restrictions on access and behaviour	20 (34)	2 845 594	56 700
Marketing regulations	Content that addresses rules and policies governing the promotion and advertising of vape products, including restrictions on targeting minors, the use of appealing flavours or packaging and compliance with legal advertising standards	4 (7%)	364 251	14 375.5
Government action	Content that highlights initiatives, policies, or enforcement measures taken by governments to address vaping‐related issues, such as implementing regulations, conducting crackdowns on illicit sales or launching public health campaigns	7 (12)	2 080 689	171 853.5
Government loopholes in regulation	Content that highlights gaps, weaknesses or inconsistencies in government regulations related to vaping, which allow for the bypassing of laws or the continued availability of unregulated or illicit products	4 (7)	337 058	825
Alarm and advocacy				
Sensationalist messaging	Content that uses exaggerated, alarming or dramatic language and visuals to create fear, shock, or outrage; often sensationalising the risks, harms or societal impact of vaping	5 (9)	8627	151.5
Education and regulation awareness	Content that aims to inform viewers about vaping laws, regulations and the importance of compliance, including raising awareness about age restrictions, product safety standards and the risks of unregulated or illicit vaping products	14 (24)	2 017 819	18 454
Illicit and counterfeit products				
Products containing illegal substances (i.e. THC)	Content including vape products containing illegal substances such as THC	8 (14)	441 632	2117
Availability of counterfeit products	Content that highlights the widespread presence and ease of access to counterfeit products	20 (34)	2 660 404	602.5
Contents of vapes	Content referring to any ingredients or contents of a vape	10 (17)	440 893	2822.5

*Note*: The domains presented are not mutually exclusive and TikTok videos can be coded into multiple.

Abbreviation: THC, Δ‐9‐tetrahydrocannabinol.

#### Summary of results

Videos categorised under the coding sub‐domains ‘apathy towards law’ were the most common (57%), followed by ‘entertain/humour’ (50%) and ‘sub‐culture and shared experience’ (50%) (see Table 4). Videos falling under these domains had a collective like count of over 21 million.

A substantial number of videos portrayed illicit vape use in a positive light (47%) with a like count of almost 5 million. Neutral depictions of illicit vape use were of a similar prevalence (31%) with approximately 4 million likes, whereas negative depictions were much less prevalent (22%) with around 2 million likes.

Three distinct groups emerged from the culmination of aforementioned domains and codes: (1) ‘TikTok videos intended to inform and educate’; (2) ‘TikToks promoting an illicit vape subculture’; and (3) ‘Tikoks glamorising and trivialising illicit vaping behaviours'. These were identified as the most prominent overarching categorisations of the content. The three groups were explicitly distinguished by perceived content intention: to inform, to promote the behaviour or to downplay and romanticise it. Although these groups are presented distinctly, they are not mutually exclusive, with videos often belonging to multiple groups.

##### TikTok videos intended to inform and educate

This group was formed from a large proportion of the content that aimed to provide viewers with factual information on the dangers of illicit vaping. Typically, videos within this group adopted an educational approach to raise awareness and inform the audience on the issues surrounding illicit vapes such as health risks and harmful ingredients.

Despite comprising a smaller proportion of the sample, ‘inform and educate’, videos still garnered substantial engagement, with approximately 2 million total likes. Comparable engagement was observed for ‘availability of counterfeit products’ and the ‘health concerns of illicit vapes’ (4 million collective likes), whereas videos addressing legislation, age restrictions and government action received 7 million likes.

**FIGURE 3:**
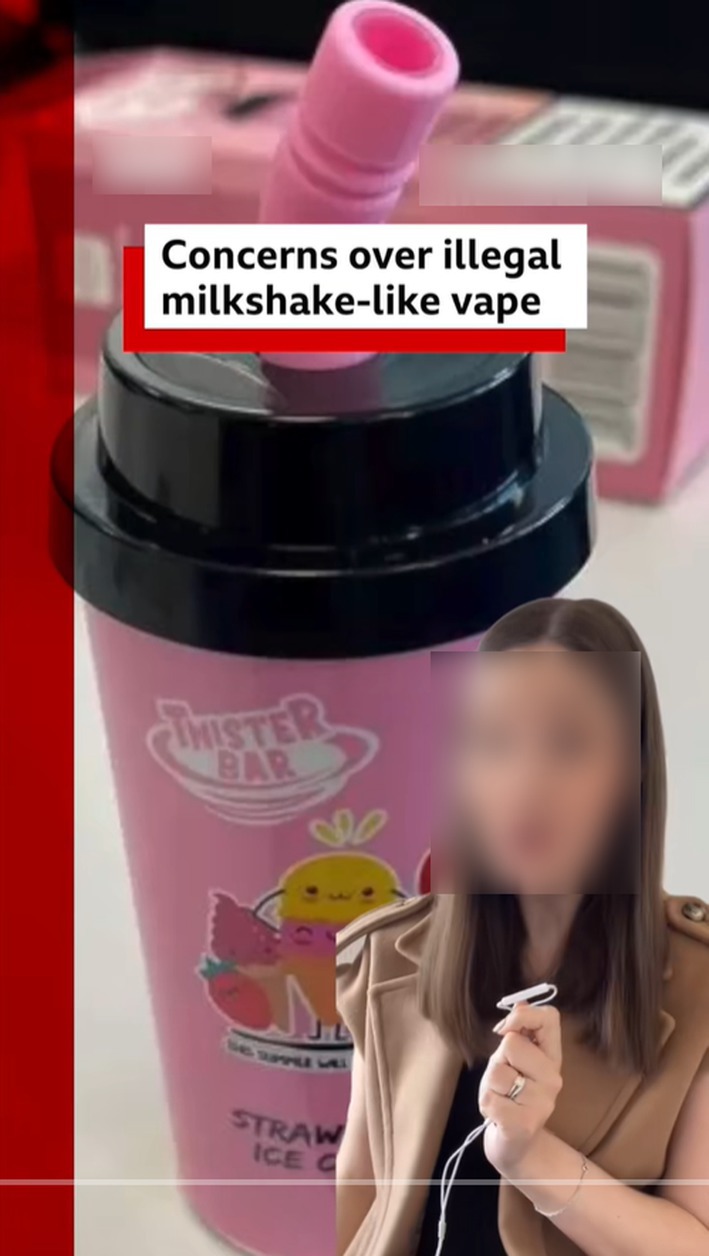
Example of a United Kingdom (UK) news report TikTok.

**FIGURE 4:**
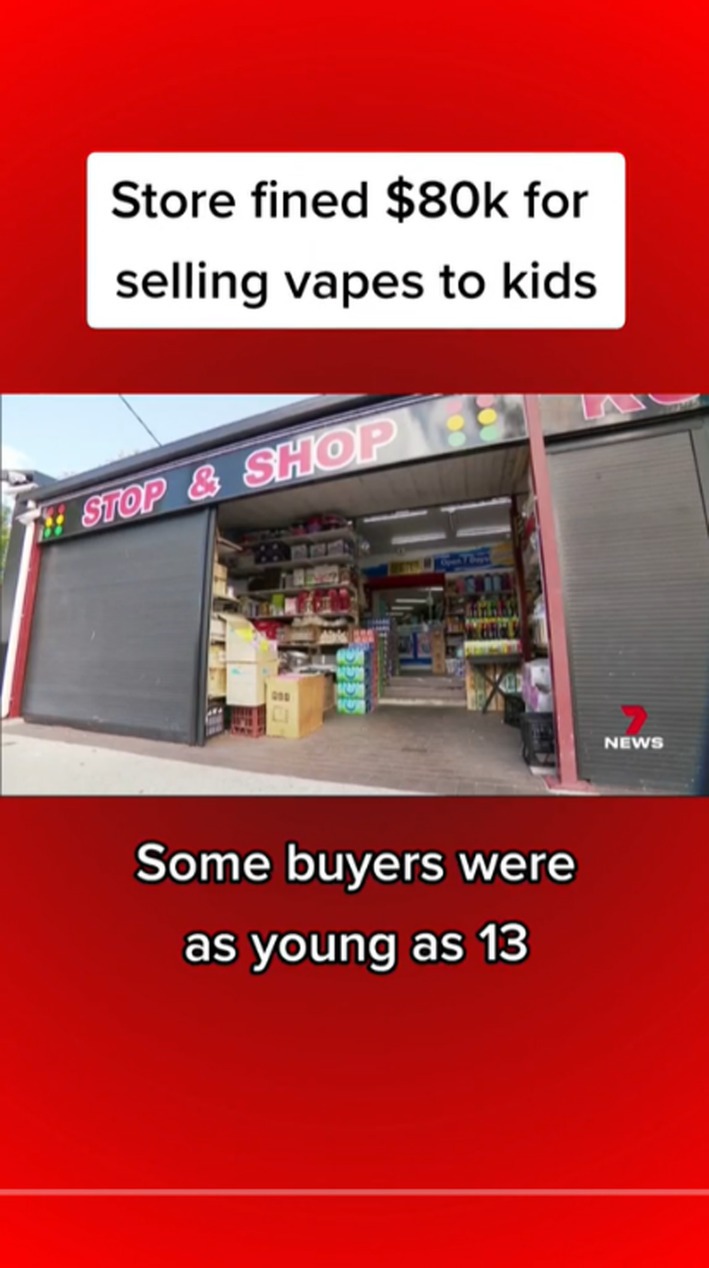
Example an Australian news report TikTok.

**FIGURE 5:**
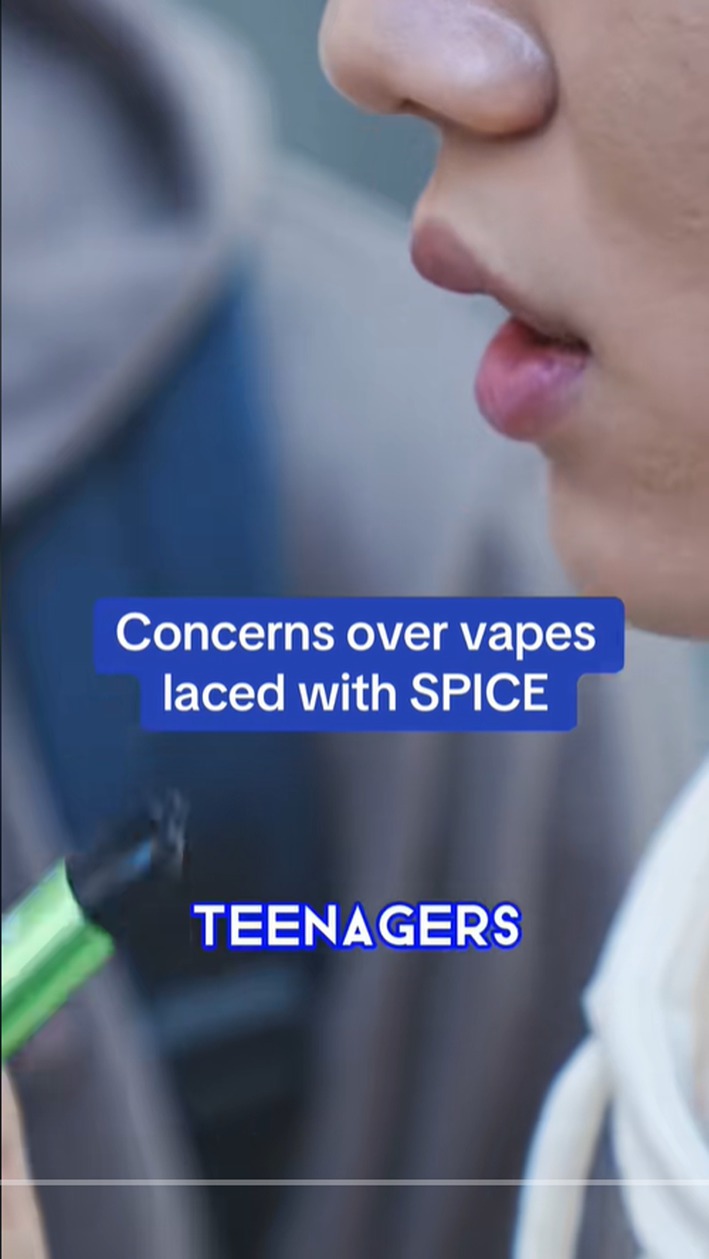
Example of a United Kingdom (UK) news report TikTok.

Typically, these videos appeared in a news report format and often outlined the key issues surrounding illegal vaping among young people, including the ease of access, illegal tank sizes, harmful ingredients and regulatory oversight. Many communicated this information in a sensationalist tone, often accompanied by dramatic audio to create a sense of foreboding. These videos tended to display the most extreme examples of illicit vape activity such as showing the most novel‐looking products (Figure 3), focusing on the youngest buyers (Figure 4) and highlighting vapes contaminated with synthetic cannabinoids (Figure 5).

##### TikToks promoting an illicit vape subculture

**FIGURE 6:**
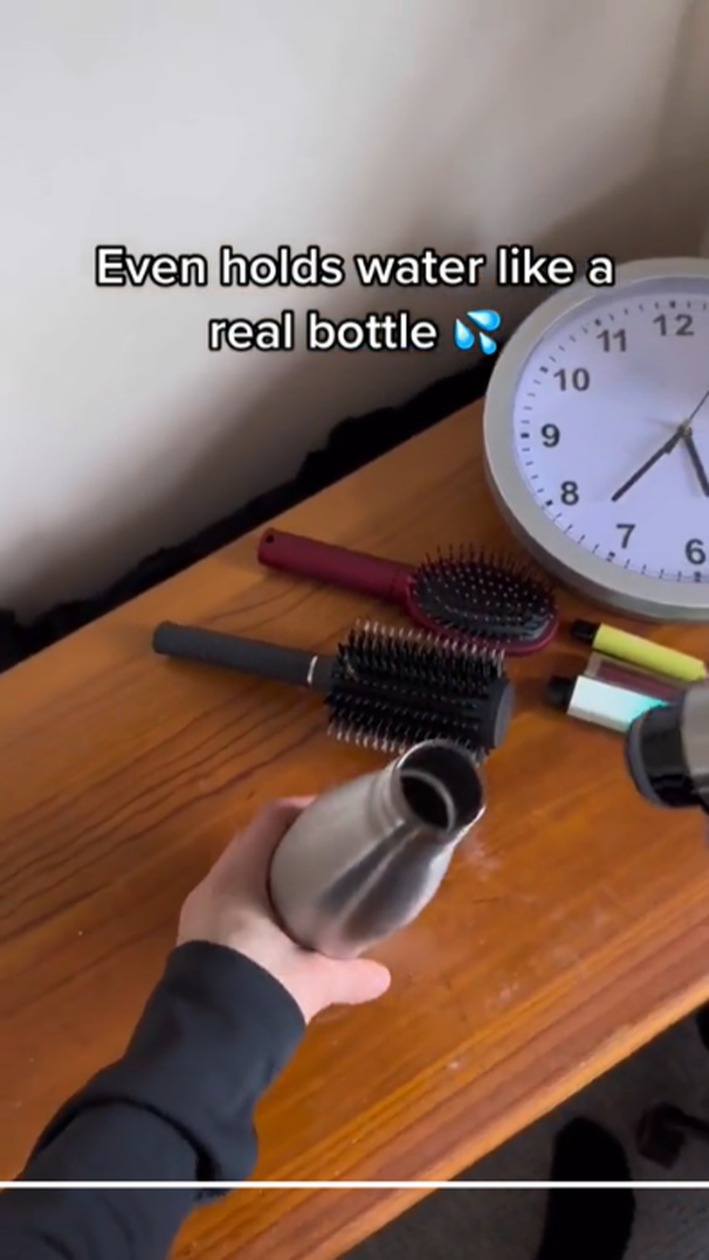
Example of a TikTok promoting a vape container.

These videos typically fostered a sense of community among viewers, through framing vaping as part of a rebellious lifestyle and glorifying the act of evading regulations. The celebration or endorsement of an illicit vape subculture was prominent within the sample of videos. This often‐involved video creators using their platform to share tips on accessing unregulated or counterfeit products, endorsing methods used to circumvent laws or hide activity from authorities or parents and displaying apathy toward vaping laws. For example, one video promoted a water bottle device manufactured specifically with a compartment to hide vapes in (Figure 6).

A high proportion of videos (76%) appeared under the theme of concealment and accessibility. Specifically, 48% of the TikTok videos had content relating to both the ease of access and the underage sales/purchasing of vapes. Together, these garnered a collective like count of over 9 million. In addition, 40% of videos included vape concealment and evasion strategies and were liked almost 2 million times.

**FIGURE 7:**
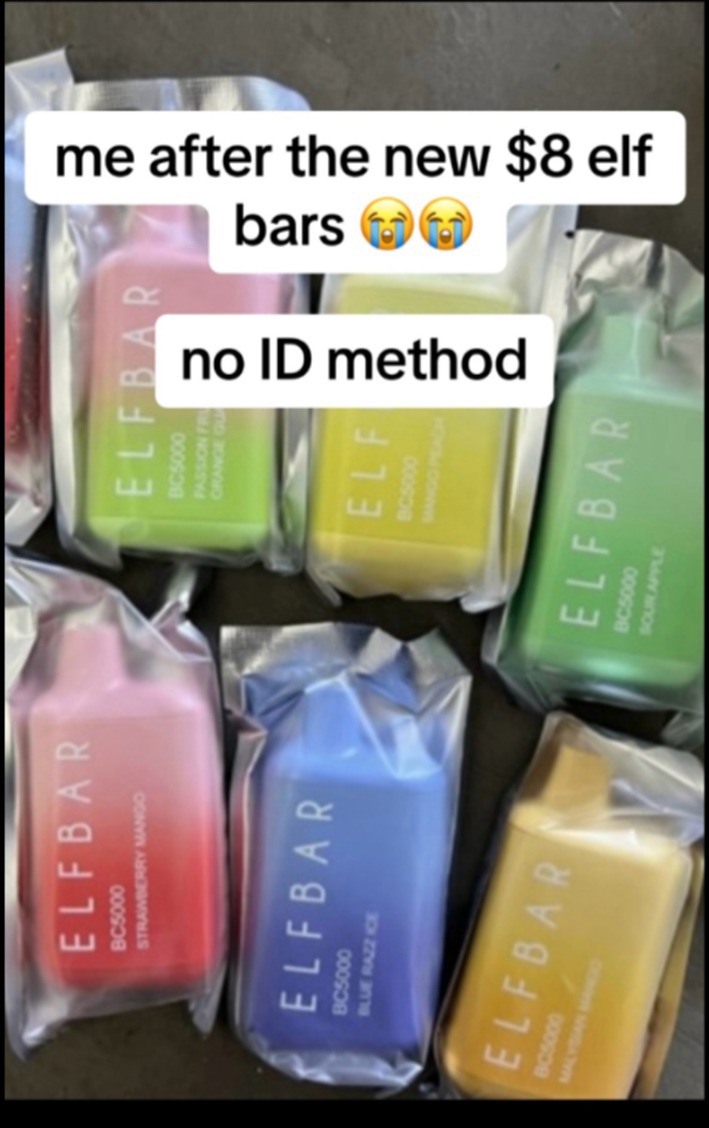
Example of a TikTok promoting no ID vapes.

**FIGURE 8:**
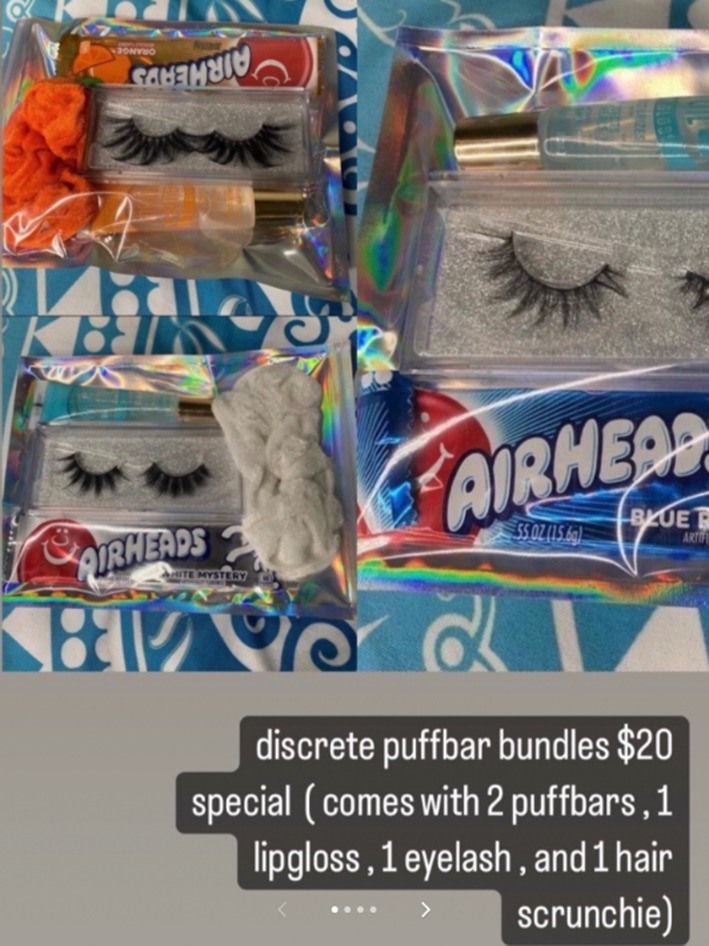
Example of a TikTok promoting a ‘puff bundle’.

The endorsement of an illicit vape subculture often coincided with ‘appealing marketing’ methods (24%), whereby attractive colours and absent age verification enhanced the allure of the vaping lifestyle (Figure 7). Moreover, vapes were often discreetly promoted in bundles, with alternative products such as cosmetic and confectionary items (Figure 8). The ‘discreteness’ within these videos often contributed to the illicit vape subculture through promoting a sense of camaraderie and reassuring people that they will not be caught for their illegal activity. Such camaraderie was further reinforced through videos frequently being paired with upbeat music.

Another prevalent theme was ‘social and cultural perceptions’ of illicit vaping. Within this, the sub‐domains of the ‘normalisation of vaping’ (40%; 5 million likes) and the ‘satisfaction from rule breaking’ (36%; 24.5 million likes) fed into a wider perception of vaping as a socially acceptable or even rebellious act. ‘Satisfaction from rule breaking’ as a standalone sub‐theme received the highest engagement levels with over 24.5 million cumulative likes, therefore, indicating that young audiences derive a sense of empowerment stemming from activities that defy societal norms.

**FIGURE 9:**
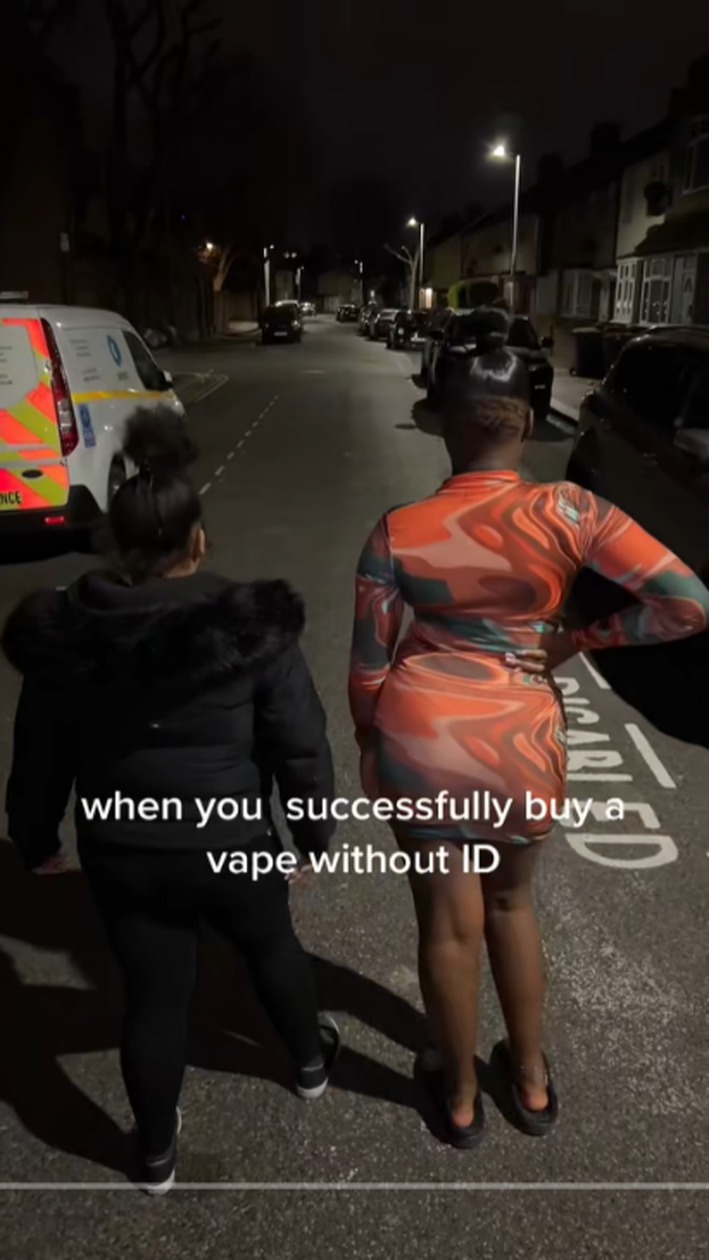
Example of a TikTok celebrating underage purchases.

**FIGURE 10:**
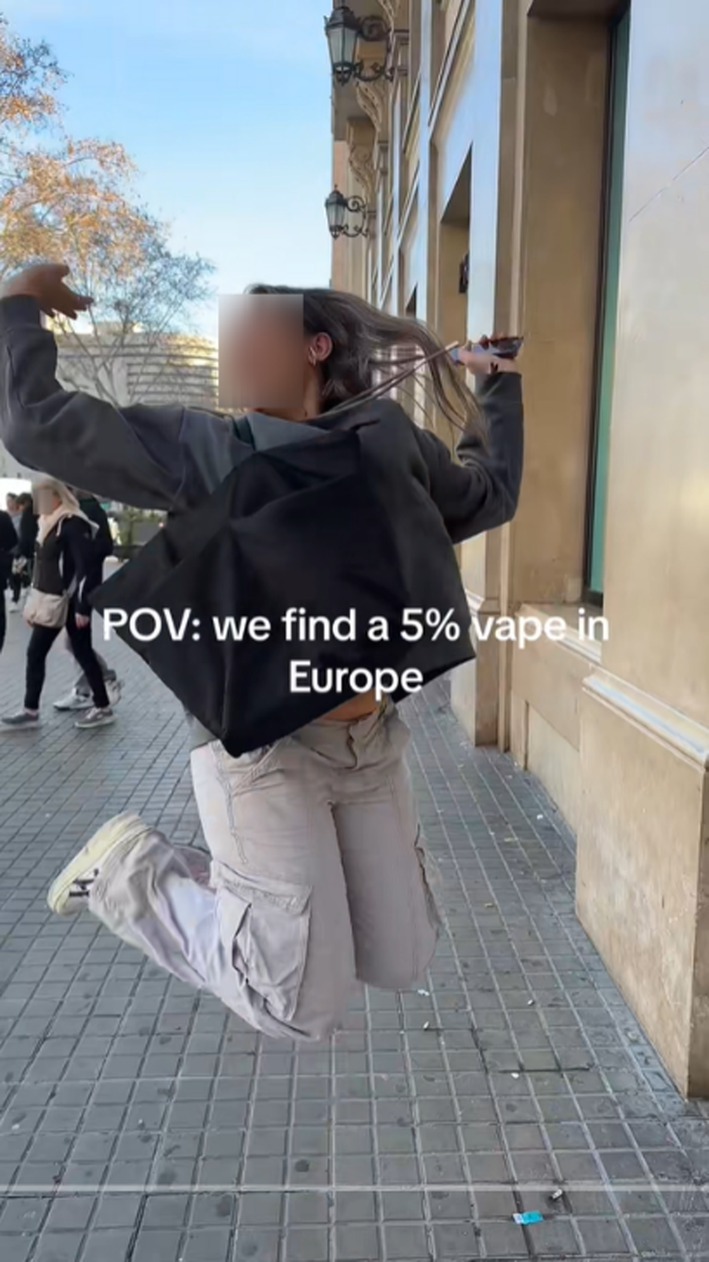
Example of a TikTok celebrating illegal purchases.

Apathy toward the law formed a key component of the illicit vape subculture, with a multitude of videos showcasing the flaunting of legal boundaries and defiance of regulations as integral to the wider appeal of vaping. Specifically, 57% of videos belonged to the ‘apathy towards law’, receiving over 6 million likes. As a stark contrast to the informative videos, these appeared ‘homemade’ and were often paired with upbeat audio to provide an element of entertainment. One such video depicted two young people celebrating a successful underage vape purchase (Figure 9), while another displayed two individuals expressing joy at acquiring a 5% nicotine vape in Europe, where such strength is prohibited (Figure 10).

##### TikToks glamorising and trivialising illicit vaping behaviours

**FIGURE 11:**
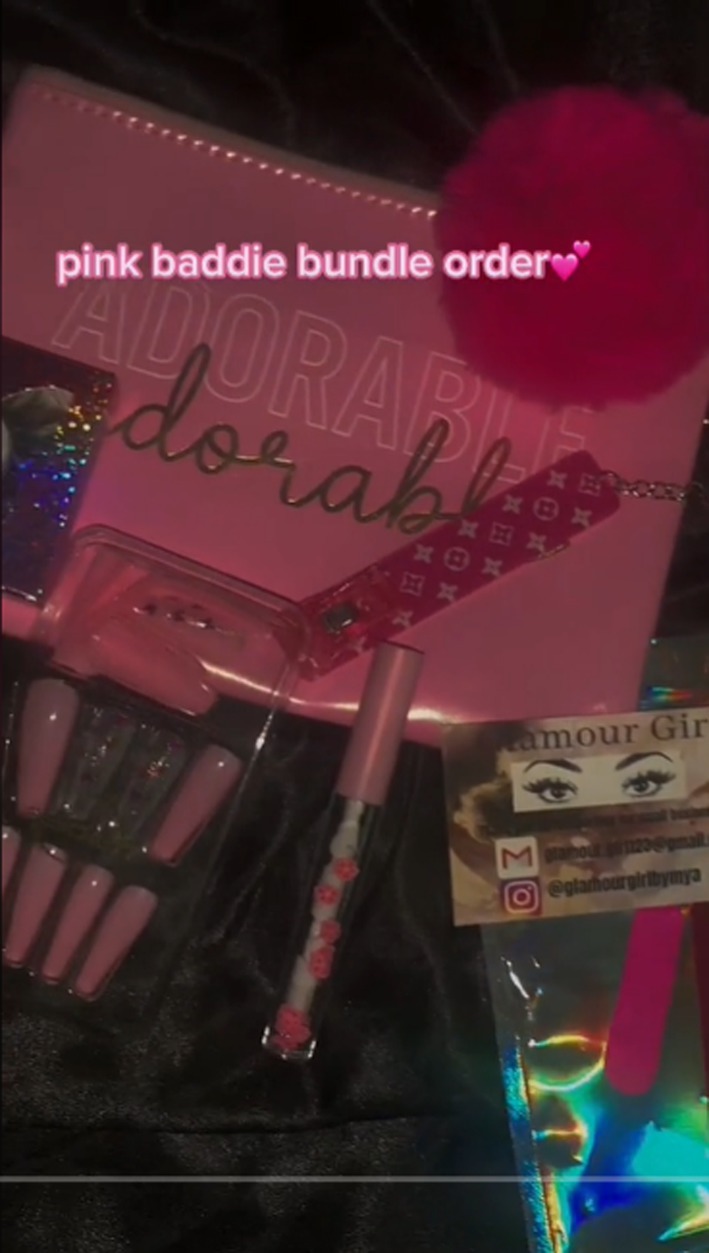
Example of a TikTok promoting a ‘pink baddie’ bundle.

This classification emerged from a multitude of videos presenting illicit vaping in an appealing or light‐hearted manner, using visuals and humour to downplay associated risks. The aesthetics and ‘thrill’ of illicit vaping formed the primary focus of the content. Beyond normalising the illicit vape subculture, several videos romanticised or glamorised illicit vaping (36%; 746 305), trivialising any potential health risks. Specifically, the visual and aesthetic appeal of illicit vaping was a prominent feature within many of the videos. As such, illicit vaping behaviours were depicted as a facet of glamour and often featured in videos promoting fashion or cosmetics. For instance, a proportion of videos were dedicated to integrating vape products into the beauty realm, with several marketing vapes as a product to be discreetly contained within cosmetic packages. These often used casual and ‘trendy’ language used in a youth context such as ‘pink baddie bundle order’ (Figure 11).

**FIGURE 12:**
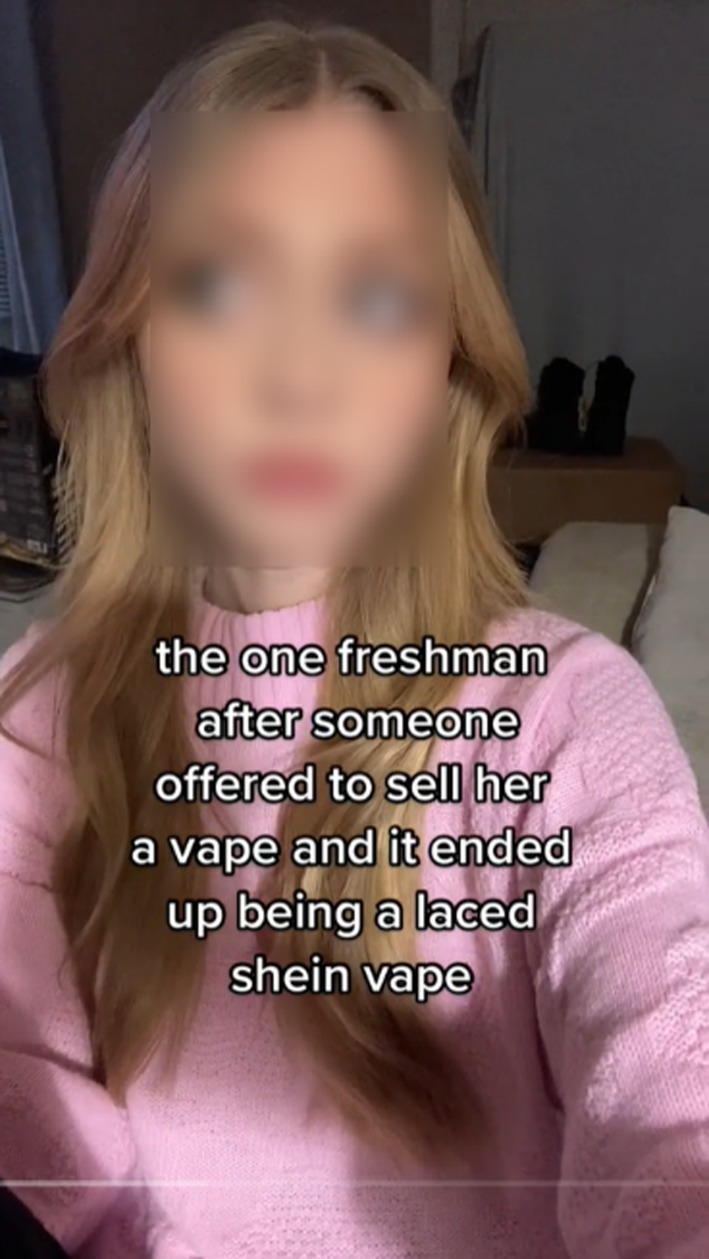
Example of a TikTok glamorising a vaping experience.

**FIGURE 13:**
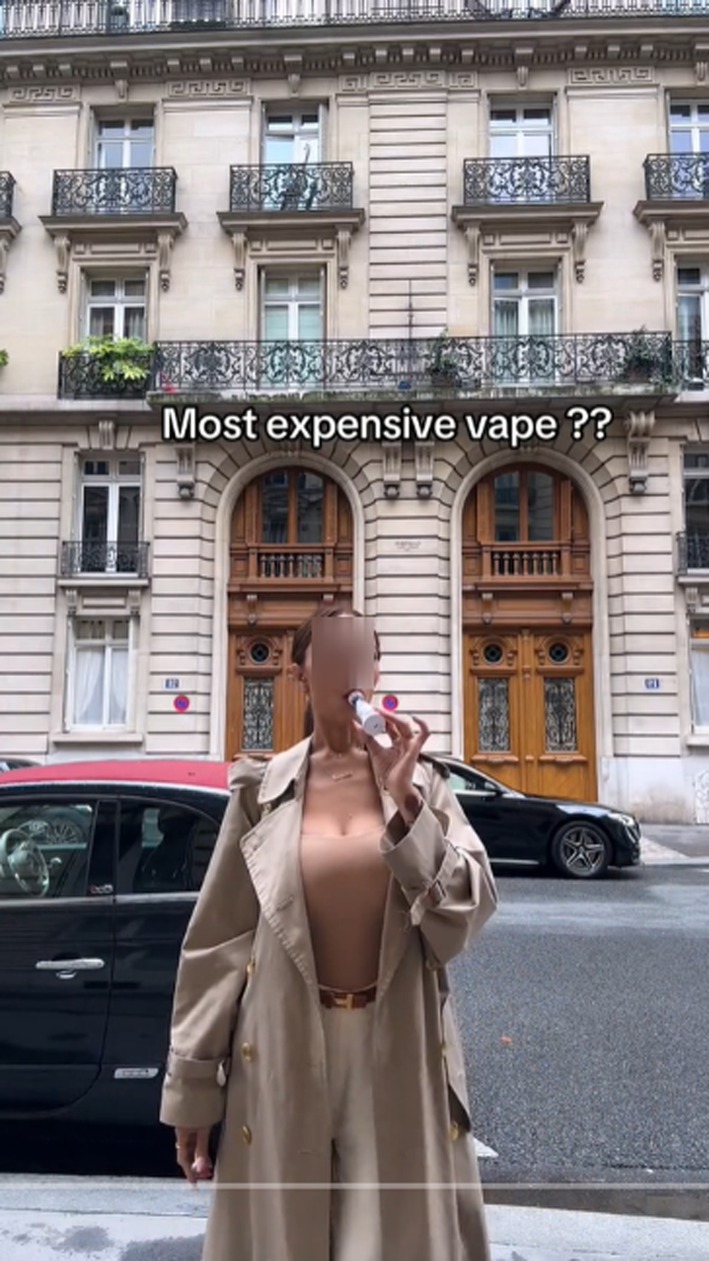
Example of a TikTok glamorising the ‘vaping aesthetic’.

The glamorised element of vaping was often presented via the actor or model central to the video. For example, one video appeared to sensationalise an experience within the illicit vape trade by featuring a glamorised actor and accompanying this with an audio of a pop song (Figure 12). In a further example, a female presenter displayed an illegal vape as a fashion accessory, framed ostentatiously as part of her ‘chic’ aesthetic (Figure 13).

## DISCUSSION

This study systematically analysed illicit vape content in two divergent streams of information sources viewed by young people: online educational resources and TikTok videos. Our analysis revealed stark differences between educational resources and TikToks in terms of their depiction of illicit vaping, varying in both their content and communicative tone. The quality assessment of educational resources revealed high authorship and accurate information depiction, yet information on illicit vaping lacked comprehensive coverage and was often presented merely as an ‘add on’. Educational resources tended to focus on the vaping phenomenon more generally and omitted detailed information on illicit vaping and its health impacts. Furthermore, resources lacked appeal to young people, with information presented in disengaging formats and adopting a formal tone when referencing illicit vaping. Conversely, TikToks in this study predominantly adopted a pro‐vaping stance in which illicit vaping content was deemed a normalised feature of young people's media.

The educational resource quality assessment indicated strong performance in the ‘social responsibility’ domain, with the majority signposting the reader to further resources and support services. However, the quality was compromised through a lack of youth relevance. Resources frequently presented illicit vaping information in disengaging formats, such as large walls of text, often accompanied by moralistic health messaging. Therefore, although the resources can have an impact through their appropriate signposting and accuracy of information, they might not resonate with young people because of limited accessibility and relevance. This aligns with evidence that adolescents prefer unambiguous language surrounding vaping harms [35]. Similarly, research evaluating vaping prevention campaigns found that overly factual campaigns failed to resonate with young people, with participants expressing the need to identify with the vaping experience [36]. Emerging communication channels such as TikTok, which often incorporate personal narratives, might, therefore, represent a more promising communicative platform for disseminating information on illicit vaping and reaching wider audiences.

Despite having similar intentions to ‘educate’ people about illicit vaping, TikTok videos intending to ‘inform and educate’ typically adopted a more sensationalist tone in presenting the most extreme reports and anecdotes. For example, videos in a news‐report format were often accompanied by dramatic audio‐visual effects. Although this approach appears successful in capturing attention, it risks distorting perceptions and overshadowing more balanced, informative discussions of illicit vaping. This is concerning given the growing evidence that young TikTok users are becoming increasingly accustomed to relying on third‐party social media apps as their primary source of discovering novel information [25].

Because the demographics of TikTok users were not examined, the high engagement with the ‘inform and educate’ videos may reflect older populations (i.e. parents) predisposed to more ‘anti‐vaping’ attitudes. This is plausible given that research suggests that the use of fear in health claims has limited resonance with young people [37], while hyperbolic fear appeals have been shown to have limited implications in vaping prevention messaging [38]. Nevertheless, the significant engagement with information‐oriented content demonstrates TikTok's potential as a public health communication channel.

A large proportion of videos capitalised on the novel appeal of illicit vaping, promoting a distinct vaping subculture and shared identity. The substantial engagement garnered from videos intended to ‘humour and entertain’ aligns with literature identifying comedy as a ‘core motive’ of vaping TikToks, which are typically absent of health implications [3, 39, 40]. In a similar vein, the prevailing positive depiction of illicit vaping concurs with previous literature highlighting that most vaping‐related content on TikTok is pro‐vaping [40, 41]. For example, Basch *et al*. [39] exemplified that more than half of the sampled videos were pro‐vaping, accumulating over 74 million views. Therefore, young people are routinely exposed to content that normalises illicit vaping as socially acceptable, potentially reinforcing its appeal and influencing viewer behaviour.

The element of ‘shared experience’ and vaping subculture evident within the TikToks reinforces a wider normalisation of illicit activity. As reflected in e‐cigarette content on Instagram [42], the collective element of illicit vaping is often reinforced through hashtags such as #vapenation, #vapelife and #hiddennic, indicating a distinct shared experience. The sharing of colours, flavours and procurement information within the videos concords with research identifying disposable vape use as a normalised behaviour within online communities [11]. Collectively, these findings suggest vaping forms an important part of young people's identity, often reinforced by peer‐driven behaviour and contributing to a culture of initiation and experimentation.

Evidence from surveys and empirical investigations has demonstrated that interpersonal stories on social media contributed to broader, normalised vaping perceptions among young people [43, 44]. The illicit vape subculture evident in the current sample has potential behavioural ramifications. Repetitive exposure to illicit vaping content contributes to its broader normalisation of vaping among receptive users, particularly when the content is presented without any risks [21]. This is consistent with the ‘mere exposure effect’ [45], whereby individuals are more likely to favour behaviours merely because they are familiar with them. This is important because research indicates that identification with pro‐vaping communities is associated with resistance to cessation and reduced behavioural control over vape use [46]. Therefore, continuous exposure to illicit vaping content may further perpetuate the normalisation of illegal activity, while simultaneously making it more difficult for young people to disengage with their vaping habits.

In addition to the normalised illicit vape subculture, TikTok videos included content promoting the ‘concealment and accessibility’ of vapes. Many videos within this theme were intertwined with ‘underage sales and marketing’ content, often with a positive sentiment and encouraging apathy toward the law. This corroborates previous research suggesting that the positive promotion of e‐cigarettes on TikTok can engender addiction apathy, with young people expressing minimal concerns for long‐term health consequences [15, 47]. Regulations appeared to have limited impact on behaviour, with many videos showcasing illicit activity and defiance in a positive light. Such indifference to regulation extends previous work, which found that young people often engage in ‘stealth vaping’, in which devices are used discreetly inside locations where vaping is forbidden [11]. Therefore, rather than existing as a deterrent, the threat of prohibitive policies and punitive outcomes contributed to the ‘thrill’ and ‘excitement’ of undertaking illicit activity.

The presence of ‘self‐proclaimed’ business owners promoting vapes through discreet methods extends previous findings identifying several ‘underground’ promotional techniques frequently used to target youth [48]. Specifically, TikTok vendors often circumvent regulation through marketing vapes as part of cosmetic ‘puff bundles’, which evade age verification and permit easy access to unregulated vaping products. Videos in this study reflected young people's ability to easily bypass restrictions and seek out accounts that sold ‘better value’ illegal devices with no requirement to provide identification. This concords with evidence that young people are not always aware of regulations, and they are able to exercise their local knowledge and digital prowess to seek out ‘permissive’ businesses who support their needs [11].

Furthermore, the integration of glamour as a core facet of illicit vape promotion aligns with research linking the ‘puff bar aesthetic’ with the appeal and accessibility of underage vaping [15]. Disguising the sales of vape products through cosmetic packaging reflects the nuanced ways in which TikTok can be used to promote activities that circumvent platform guidelines and target specific audiences. Through blurring the lines between covert advertising and legitimate product promotion, these videos diminish the perceived harm of engaging in illicit activity through their trivial and ‘light‐hearted presentation’.

### Implications

Educators should capitalise on the engagement potential of video‐based social media working with young people themselves, to disseminate evidence‐based information on illicit vaping. Traditional educational resources may now hold limited relevance to young people, meaning adopting more novel approaches such as TikTok exists as a more promising avenue. The present findings suggest that effective educational content should incorporate evidence‐based information, engaging visuals and humorous sentiment. The noticeable indifference toward legislation among the videos, along with the PPIE verification that top‐down health messaging in educational resources is unhelpful, underscores the need for more youth‐driven co‐produced approaches. Co‐production exists as a critical opportunity to empower young people in their own health literacy, while ensuring that the information they are receiving remains both relevant and accessible. Combined with more robust advertising policies prohibiting illicit activity on TikTok, such approaches may be instrumental in denormalising youth vaping and mitigating the illicit vape culture.

### Strengths and limitations

The strengths of this study include a robust and systematic approach to searching and analysis with independent double coding, third reviewer conflict resolution and PPIE verification. Involving a PPIE representative with lived vaping experience enhances validity by offering a unique first‐hand insight into youth culture and the content that resonates with young people.

Limitations include that some of the videos (*n* = 11) were taken down before double coding occurred and so were second coded based on a textual summary of the video, potentially lacking the depth of full video analysis. However, this omission was deemed inappropriate given their substantial reach and engagement. The small sample size of both the educational resources and TikToks exists as a limitation of this study yet highlights a lack of available information on illicit vaping. UK‐centric searches limit the generalisability of educational resource findings and because of the global distribution of content on TikTok's algorithm, videos may not exclusively represent UK‐produced global content. This study was conducted before the disposable vape ban in 2025, meaning the findings reflect the media environment before this implementation and may not capture the evolving landscape of internet content following these changes.

## CONCLUSION

Both educational resources and TikTok videos demonstrated an overall paucity of engaging, evidence‐based information on illicit vaping. Such information sources were juxtaposed in their content. Educational resources typically adopted a formal, authority‐driven approach, whereas TikTok videos presented illicit vaping in a casual and entertaining manner. The most engaging TikTok content promoted apathy toward the regulation, had a humorous sentiment and contributed to a shared ‘underground’ illicit vaping subculture. These findings underscore the need for youth‐informed educational resources, with young people repositioned as experts in the co‐production of impactful and relevant content.

## AUTHOR CONTRIBUTIONS

**Eleanor Bray:** Methodology; writing—original draft; writing—review and editing; project administration; investigation; formal analysis. **Sarah Gentry:** Validation; supervision; formal analysis; writing—review and editing; methodology. **Anna Varley:** Validation; formal analysis; writing—review and editing. **Caitlin Notley:** Conceptualization; writing—review and editing; supervision; methodology; project administration. **Samuel Webb:** Validation. **Emma Ward:** Conceptualization; writing—review and editing; validation; project administration; supervision; formal analysis; methodology.

## DECLARATION OF INTERESTS

None.

## Data Availability

The data that support the findings of this study are available from the corresponding author upon reasonable request.
